# Tail Posture as an Indicator of Tail Biting in Undocked Finishing Pigs

**DOI:** 10.3390/ani9010018

**Published:** 2019-01-08

**Authors:** Torun Wallgren, Anne Larsen, Stefan Gunnarsson

**Affiliations:** Department of Animal Environment and Health, Swedish University of Agricultural Sciences (SLU), PO-Box 234, 532 23 Skara, Sweden; Anne.Larsen@slu.se (A.L.); Stefan.Gunnarsson@slu.se (S.G.)

**Keywords:** tail docking, animal welfare, swine, fattening pig, tail damage

## Abstract

**Simple Summary:**

Tail biting is a large welfare problem in modern pig production, causing pain and reduced health and production. The identification of tail biting is important for minimising the risk of the escalation of the behaviour and its consequences. Tail posture (i.e., tail hanging or curled) has been suggested to depend on the presence of tail wounds and, therefore, has been suggested as an indicator of tail biting. This study investigated the relationship between tail position and tail damages at feeding, since that could be a feasible time for producers to detect tail posture. The experiment showed that 94% of the pigs had curly tails and that pigs with wounds were more likely to have hanging tails than pigs with nondamaged tails. By observing the tail position at feeding, we were able to identify pigs with tail wounds in 68% of cases simply by scoring pigs with hanging tails. To conclude, the scoring of pigs with hanging tails at feeding was found to be a useful tool for identifying tail damages, which may otherwise be difficult to detect by the caretaker.

**Abstract:**

Tail posture (i.e., hanging or curled) has been suggested to be an indicator of tail biting, and hanging tails predisposed to damage. The aim of this study was to investigate if tail posture was feasible as a tail damage indicator in a commercial setting. The study was carried out on one batch of 459 undocked finishing pigs (30–120 kg in weight). Weekly scoring of tail posture was combined with the scoring of tail lesions. Tail posture was observed at feeding to facilitate the usage of the method in commercial settings. A curly tail was observed in 94% of the observations. Pigs with tails scored with “wound” were 4.15 (*p* < 0.0001) times more likely to have hanging tails, and pigs scored with “inflamed wounds” were 14.24 (*p* < 0.0001) times more likely to have hanging tails, compared to pigs with nondamaged tails. Tail posture correctly classified tails with “wound” or “inflamed wound” 67.5% of the time, with 55.2% sensitivity and 79.7% specificity, respectively. The method of observing the tail position at feeding seems useful as a complement to normal inspection for detecting tail biting before tail wounds are visible to the caretaker.

## 1. Introduction

Tail biting is a well-known issue within European pig production. In this context, tail biting is defined as one pig orally manipulating another pig’s tail, and the phenomenon occurs in both docked and undocked pig populations [1]. Tail biting and subsequent tail injuries are known to cause stress and reduce welfare in both the injured and biting pigs [2]. Additionally, tail wounds may reduce weight gain and cause condemnation of the whole or parts of the pig carcass. Therefore, the prevention of tail biting is important for profitability, as well as the improvement of animal welfare. Although both legislative and consumer demands require pigs to be raised without tail docking [3], the most recent survey published by the EFSA council (2007) shows that 90–95% of the pigs produced within the EU are tail docked to reduce the risk and consequences of tail biting [1,4,5]. Raising undocked pigs has been associated with an increased risk of tail biting, and therefore management routines for raising pigs with intact tails must be developed before realistically eliminating tail docking within the EU [4,6].

Even though a lack of occupation has been found to be one of the major risk factors for developing tail biting in pigs [1], the causal background of tail biting outbreaks is complex and has not yet been fully understood. It has been suggested that tail biting is a redirected behaviour with its background in unfulfilled exploratory behaviour [7]. Nevertheless, tail biting is multifactorial, with several other factors having an impact on the development of tail biting, such as genetics, feed type and indoor climate [2,8]. It is hard to stop an outbreak once tail biting has developed [9]. Fraser [10] found that pigs show an increased attraction to chewing the tails of other pigs when blood is present, although this attraction to blood is highly individual. This phenomenon is considered part of the explanation why small tail wounds may lead to large tail biting outbreaks within a short period of time [10].

Since tail biting outbreaks may escalate rapidly and are difficult to stop, the emphasis must focus on minimizing the risk factors of outbreaks. It has been hypothesised that if tail biting behaviour is detected early, i.e., before severe tail wounds appear, a change in management might inhibit an outbreak. Tail biting behaviour is usually not detected until tail lesions are present, which increases the difficulties in stopping outbreaks [11]. Tail posture has previously been suggested to be an indicator of tail biting in pigs, with researchers hypothesising that affected pigs should be more prone to have their tail in a hanging posture than unaffected pigs [12,13].

Already in 1990, McGlone et al. [14] noted pigs having uncurled tails when tail biting occurred. Tail posture has been suggested to be a protective measure as well as being part of pig communication and comfort [12,14]. For example, heat-stressed pigs may be more likely to have hanging tails, whereas pigs being handled by a familiar person, experiencing positive pig comfort, might have curled tails [12]. In weaned piglets, it has been shown that tail posture is related to tail lesions and that hanging tails may be a predictor for tail lesions occurring 2–3 days later [11]. According to Lahrmann et al. [15], hanging tails were more common in pigs kept in pens where tail biting had been observed than on pigs in pens where no tail biting had been observed. They also found that the number of pigs with hanging tails observed on one day was correlated to the number of tail lesions observed the day after. A recent study by Larsen et al. [13] found that a tucked-in tail increased the odds for tail wounds on the same day in finishing pigs. However, their method was found to give many false identifications. Therefore, we hypothesised that tail posture could be a suitable option for the early detection of tail biting under commercial conditions due to the ease of recognition of curled or hanging tails.

The aim of this study was to investigate the relationship between tail posture (hanging or curled) at feeding and tail lesions in finishing pigs. The hypothesis was that pigs with hanging tails were predisposed to having their tails bitten and had tail lesions that could be assessed with closer visual examination. It was further hypothesised that tail posture would be easily detectable at feeding, which could then be used as an intervention method at commercial farms. Furthermore, we attempted to determine the specificity and sensitivity of tail posture observation in relation to the scoring of tail lesions.

## 2. Materials and Methods

The study was carried out at a commercial farrow-to-finish pig farm in southwestern Sweden for a total of 102 days. A total of 14 observations were carried out (one per week) from December 2017 to March 2018. One batch of 458 pigs was studied from approximately 30 kg live weight (LW) until slaughter (approximately 120 kg LW). Pigs were sent to slaughter in five shipments based on LW, and the trial was ended when the majority (i.e., >70%) of the pigs had been sent to slaughter, on week 14 (Table 1). All pigs were the progeny of crossbred sows, Landrace*Yorkshire (TN70) and Hampshire boars. All sows were kept in loose-housed farrowing systems with straw. All pigs were undocked and males were surgically castrated by two incisions made in the scrotum during the first week of life after receiving analgesic treatment. The analgesia was performed with an injection of 0.3–0.5 mL/testicel of lidocaine 20 mg/mL and adrenalin 0.036 mg/mL (Lidokel-Adrenalin vet.^®^). The piglets were weaned at approximately five weeks of age.

When moved into the finishing pig stable, pigs were sorted by size (largest pigs kept together and smallest pigs kept together) but not by sex. The 458 pigs studied were allocated into 42 pens, which housed either 10 (n = 4), 11 (n = 37) or 12 (n = 1) pigs per pen. The pens each had an area of 10.49 m^2^, 7.81 m^2^ of which was solid floor and 2.68 m^2^ slatted floor. In each pen there was a 3.4 m-long feeding trough at the long side of the pen and a nipple drinker above the slatted area. The pigs were fed liquid feed with an automatic feeding system according to the farm feeding regime of four meals per day until week 12, when the feeding regime was changed to three meals per day. The pigs were inspected daily and the pens were manually cleaned and provided with fresh chopped straw once a day (~25 L of straw provided on the floor or ~44 L provided in a straw rack; 25 L of straw weighs ~1.8 kg).

The pigs were not individually tagged; therefore, in order to keep track of individuals, they were marked with spray paint (PORCIMARK marking spray, Kruuse, Denmark) twice per week. The animals were marked with one to three lines on their back in red, blue or green. One pig per pen was kept unmarked.

The tails of the pigs were scored weekly by palpation with regard to tail shortening, tail damage, and wound freshness, according to the scoring protocol presented in Table 2. Pigs that were observed to be limping severely or unwilling to stand or put weight on at least one of their limbs at scoring were recorded as lame. All scoring was carried out by the same operator.

The tail posture of all pigs was scored weekly on the same day as the lesion scoring. A tail was considered to be curled if the major part of the tail was curled and pointing upwards in relation to the horizontal extension of the back; otherwise the tail was scored as hanging. Tail position was recorded at feeding by filming. Pigs were filmed when all pigs were standing at the feeding trough. The tail position for each pig was then scored by observation of the video recordings. If a pig’s tail was not observed during filming, due to the pig sitting down or not visiting the feeding trough, tail position was recorded as missing. Pigs whose tails were so short that the tail position could not be determined were also scored as missing. Filming the entire stable took on average 9.8 min.

Statistical analysis was carried out using the following three software tools, StataIC 15.1 (StataCorp LLC, College Station, TX, USA), MLwiN (Centre for Multilevel Modelning, University of Bristol, Bristol, UK) and SAS 9.4 (SAS Institute, Cary, NC, USA). Data on tail position, tail lesions (length, damage and freshness) and lameness was collected on individual pig level at each sampling occasion. Rows with missing data were removed from the data set. The data in this study was hierarchical with three levels consisting of Pen (highest), Pig and Time (lowest). Each pig was observed at several time points (Time) and hence repeated measurements had to be taken into account.

This was an observational study to investigate the association between tail lesions and tail posture at feeding. A binomial multivariate regression model (trend model) with tail position as the outcome was built in StataIC and MLwiN using the “melogit” command. The statistical unit was pig. Since the recordings of length, damage and freshness are closely correlated (e.g., freshness score equivalent to or more severe than crust was only given to pigs with a damage score of “wound” or “inflamed wound”), only damage was included in the final model. Damage was considered the most suitable choice since it had more variability compared to length and freshness. The analysis included the following independent variables; Pen, Pig, Lame (yes/no), Damage (nondamaged/swollen/bite marks/wound/inflamed wound), Sex (gilt/barrow) and Time (1–14). Time was standardised using the following equation: $sTime=\frac{Time-7.5}{6.5}$ (Time ranging from 1–14) to receive values of sTime ranging from 0 to 1. The relationship between the data and Time was found not to be linear, and therefore Time^2^ was used to allow a better linear fit to the data. All variables were included in the model, which was subsequently reduced by backward selection of the significant variables (*p* ≤ 0.05). Time and Time^2^ were kept in the model to account for repeated measurements.

Clustering within pens and pigs was accounted for by including random slopes for sTime at Pen and Pig level and for sTime^2^ at Pen level. The software was unable to fit a random slope for sTime^2^ at Pen level. The estimated log likelihood difference between the chosen model and the model with random sTime^2^ slopes also at Pen level was −18.8614, showing that the model without the random slope at Pen level was better. Furthermore, the analysis of variance showed that there was more variation at pig level than at Pen level, and thus the finalised model without random slope at Pen level was determined to be satisfactory.

The final model was

(1) $$\begin{array}{ll} TP_{\pi Time,Pig,Pen}= & \beta_{0ID,Pen}+Swollen_{Time,\;Pig,\;Pen}+Red_{Time,\;Pig,\;Pen}+\\ & Wound_{Time,\;Pig,\;Pen}+Inflamed\;wound_{Time,\;Pig,\;Pen}+\\ & Sex{\left(barrow\right)}_{Pig,\;Pen}+\beta_{5ID,Pen}sTime_{Time,Pig,Pen}+\\ & \beta_{6ID,Pen}sTime^{2}{}_{Time,Pig,Pen}+e \end{array}$$

To investigate the possibility of using tail posture as an indicator of tail biting and to estimate the specificity and sensitivity of the method, a receiver operating characteristic (ROC) analysis was conducted between tail posture and tail damage. A curled tail posture was considered to be either present or absent (hanging tail posture). To create the ROC curve, tail position was used to classify tails as Damaged (nondamaged/swollen/bite marks/wound/inflamed wound).

This study comprised only behavioural observations and the clinical scoring of commercial pigs. It was part of a larger trial aimed to improve animal welfare by investigating different ways of providing pigs with straw. Due to the low severity of the treatment the study did not require approval by an ethical committee according to the legislation. All pigs were managed and treated by the staff at the farm according to the ordinary management routines, i.e., injured pigs received appropriate medical treatment, such as the removal of bitten pigs and treatment with antibiotics in the case of severely inflamed wounds or reduced health status.

## 3. Results

Out of the 5713 observations of pigs scored with curled tails, 55.4% had nondamaged tails, 10.7% had swollen tails, 13.6% had bite marks on the tail, 19.6% had tail wounds and 0.8% had inflamed wounds on the tail. Out of the 379 observations of pigs scored with hanging tails, 28.8% had nondamaged tails, 7.92% had swollen tails, 7.65% had bite marks on the tail, 46.97% had tail wounds and 8.71% had inflamed wounds on the tail.

Both tail posture and tail damage on Pig level varied over time, and the number of damaged tails increased with time. The proportion of hanging tail posture fluctuated, being high in the beginning and in the end of the production period (see Table 1). The proportion of hanging tails and tail damages varied between pigs on Pen level. Two of the observed pens did not have any pigs with hanging tails during the study period, while all other pens had pigs with hanging tails on at least one occasion. Tail damage of some sort was observed in all pens during the study period. The data is provided in Table S1.

### 3.1. Model

Hanging tails were positively associated with tail damage scores of “wound” and “inflamed wound” (*p* < 0.05), however not with less severe damage, i.e., “swollen” and “bite marks”. Pigs with tail damage scored as “wound” were 4.15 times more likely to have hanging tails than pigs with nondamaged tails, while pigs with tail damage scored as “inflamed wounds” were 14.24 times more likely to have hanging tails than pigs with nondamaged tails. Barrows were 1.58 times (*p* > 0.046) more likely to have hanging tails than were gilts (Table 3).

The random slopes for sTime at Pen level and sTime and sTime^2^ at Pig level allowed for different Pens and Pigs to develop differences over time. The variation at Pig level was larger than the variation at Pen level (Table 3).

As we found that barrows were more likely to have hanging tails than were gilts, we investigated whether barrows also had more tail damage, as presented in Table 4. When comparing the tail damage of barrows and gilts in a chi-squared test, no differences were found (*p* > 0.2688).

### 3.2. Evaluation of Tail Posture as an Indicator of Tail Damage

Lowering the cut-off point of what was considered a damaged tail increased the sensitivity (i.e., the probability that a damaged pig tail was classified as damaged through tail position) of tail posture as an indicator of tail lesions (Table 5). Conversely, increasing the cut-off point of what was considered a damaged tail decreased the sensitivity of tail posture as an indicator of tail lesions. Tails scored as “swollen” had a sensitivity of 70.57%, while tails scored as “inflamed wound” had a sensitivity of 8.59%.

The specificity (i.e., the probability that an undamaged pig tail was classified as undamaged through tail position) increased when increasing the cut-off point of what was considered a damaged tail. When the cut-off point was set to “inflamed wound”, the specificity was 99.16%, while the specificity was 55.38% if the cut-off point was set to “swollen” (Table 5). The percentage of correct classifications increased with an increased cut-off point, increasing from 56.33 to 93.47% as the cut-off point passed from “swollen” to “inflamed wound”. The area under the ROC curve, corresponding to the ability to make correct classifications, was 68.62%.

When setting the cut-off point of when to consider a tail as damaged to “wound” or “inflamed wound”, the sensitivity was 55.2%, the specificity was 79.7%, and 78.1% were correctly classified. The area under the ROC curve was 67.4%.

## 4. Discussion

The finding that hanging tails were associated with more severe tail damage, i.e., wounds and inflamed wounds, indicates that a hanging tail posture does not occur until tail damage is already present. This is in line with findings by Larsen et al. [13], showing that pigs with tucked tails were about six times more likely to have a wound the same day. Hence, it does not seem to be enough to look at tail posture in this manner and frequency to identify tail biting behaviour before it has caused tail damage. However, most of the wounds detected by tail posture would not be detectable without close examination of the tail, and therefore tail posture could still be useful in commercial production for detecting tail biting before wounds are detectable from outside the pen.

The correct classification of a damaged tail through its posture was achieved in 67% of cases when the cut-off point was set to “wound”, which includes the damages that were statistically proven to be associated with tail posture by our model. This means that 33% of the pigs will be misclassified. When assessing tail posture, 44.8% of the pigs with tail damage will be missed and 20.3% will be misclassified as having wounded tails. This was evident partly from the fact that we observed pigs with hanging tails and undamaged tails (or tails with damage less serious than “wound”) while there were also pigs with wounded curly tails (ranging from “swollen” to “inflamed wound”). However, the misclassification of non-injured pigs as injured, which was also reported by Larsen et al. [13], does not have to be considered a large issue. According to a survey of Swedish farmers, the main treatment for stopping tail biting in pigs is the provision of extra straw [17]. Providing extra straw will increase rooting possibilities and could be considered as a positive enrichment while having no negative consequences. However, pigs with wounds that are missed through the assessment of tail position need to be identified through other means such as clinical examination or behavioural observations. The discovery that not only injured pigs have hanging tails is in line with the finding by Lahrmann et al. [15], that in pens without observed tail biting outbreaks approximately 15–17% of pigs had hanging tails, while in pens with observed tail biting outbreaks approximately 23–33% had hanging tails 1–3 days before an outbreak. Similarly, Zonderland et al. [11] found that around 15% of pigs with curled tails had tail damage according to our scale and that 14.6% of the pigs that had tails that were hanging or tucked in between the hind legs were undamaged. Furthermore, Statham et al. [18] also found that there were fewer pigs with hanging tails in groups with no tail biting outbreaks than in groups with tail biting outbreaks. Collectively, this shows evidence that tail posture at feeding could be used as an indicator of tail biting, even though the method has significant flaws and needs further investigation.

Compared to other studies looking at tail posture, (e.g., Zonderland et al. [11], Lahrmann et al. [15], Statham et al. [18]) the pigs in this study were scored less frequently (weekly, compared to daily or three times per week). It is possible that we could have detected tail damages at earlier stages if scoring had been performed more frequently. As found by Lahrmann et al. [15] and Zonderland et al. [11], changes in tail posture can be seen hours to days before tail lesions occur. Due to the experimental setup of our study, we were not able to detect such changes. However, there was no significant effect of time in the statistical model, indicating that time did not have a significant impact on the presence of hanging tails. Moreover, we were unable to associate damage that was less severe than “wound” with tail posture. If we had used shorter observation intervals we may have detected time dependent changes. However, our study was designed to reflect the usability for commercial farmers, who rarely have the time to observe individual pigs as frequently as once per day. We propose that this method could be incorporated into normal farm routines, for instance when farmers are checking feeding equipment or the health status of pigs.

The number of hanging tails was found to fluctuate over time; the frequency was highest at the beginning and the end of the trial period. The beginning of the production period is usually associated with stressful events for the pigs, such as being moved to a new stable, getting new pen mates and receiving new feed or a new feeding regime. Stressful events also occur at the end of the production period, when pigs are larger and the stocking density (kg/m^2^) increases. Additionally, larger pigs may be sent to slaughter, which may change the established social hierarchy in the pen, leading to fights. Moreover, some female pigs may become sexually mature, which could also alter behaviour and hierarchy in the pens. As suggested by Kleinbeck and McGlone [12], tail posture might be an indicator of pig comfort. The reason for the large amount of hanging tails observed at the beginning and end of the production period could therefore be related to stress (e.g., due to new environment or new hierarchy), rather than to tail biting. On the other hand, tail biting is also known to occur when stressors such as increased stocking density and new hierarchies arise [2]; however, the causality for hanging tails is not evident from this study.

Lahrmann et al. [15] noted that the activity of pigs influenced their tail posture, revealing that tail posture could also be a response to emotional states other than, e.g., pain or discomfort. For example, pigs engaged in rooting activities were more likely to have hanging tails compared to walking or running pigs. Furthermore, tail posture was more likely to change in a short time after pigs changed activities. As discussed by de Oliveira and Keeling [19], certain animal postures may not be specific to specific emotional states and may not be possible to assess alone but rather only in combination with whole body posture. They found that there were interactions between tail, ear and neck position and activity in cows, suggesting that cows express themselves differently during different activities (brushing, queuing for milking or feeding). In the present study we scored the tail posture at feeding. There is a lack of knowledge about how feeding alone is associated with the tail posture of pigs. From the results of this study we know that the majority of the pigs had curled tails at feeding. However, some of the curled tails had severe damage. It is possible that feeding alone makes pigs curl their tails; for example, since no other pigs are situated behind the pigs at this time, there should be no need to protect the tail between the hind legs. To completely understand the association between tail position and tail damage, the tail position should be investigated further during several different activities and emotional states. When designing the present study, the decision to score the tail posture at feeding was made for two main reasons. Firstly, it is commonly observed that most pigs have curled tails while eating. Secondly, the scoring of tail posture, especially in commercial settings, would be made easier if all pigs in a pen could be rapidly scored. When pigs in long trough pens are fed, the feeding system releases feed in one pen at the time. The next pen in line will receive feed a few seconds after the previous pen, making it possible for us to easily score tail posture on the feeding pigs. This would make it possible for farmers to follow the feeding loop and observe one pen (commonly with ~10 pigs per pen in Sweden) at a time. This assessment could then be easily incorporated into the management scheme. In this study we recorded the pigs at feeding by video in order to score the tail posture afterwards. This was performed mainly to register the ID of each pig and subsequently combine tail posture with tail scoring. In commercial farms, it would be possible merely to note in which pens there are numerous pigs with hanging tails to obtain an indication of where there could be tail biting issues. Hence, these direct observations could ease the implementation of this technique in common management practices. The time taken to observe all pens (42 pens, 459 pigs) in this trial was on average less than ten minutes at experimental setting (which is considered to be more time consuming). This suggests that this method is also feasible in systems where there are more than 10 pigs per pen as long as the animals all feed at the same time and the feeding trough is located so that the pigs eat standing next to each other, thus facilitating observation for the pig keeper.

The random slopes in the developed model took into account that different pigs may react differently. The variability was higher at individual than at pen level, suggesting that the mix of pen mates might even out individual differences. It is known that pain is a subjective experience and may cause different reactions to the same stimuli (e.g., Ison et al. [20]). Therefore, some pigs may react with a hanging tail when another pig is manipulating their tail, while other pigs might not react at all even when there is a wound on the tail. Different pigs may also have different coping strategies for avoiding tail biting. As hypothesised by Feddes and Fraser [21], a curled tail could also be a way of reducing tail biting by protecting the tip of the tail, which is the most commonly attacked part. This is converse to the hypothesis that the pig uses a hanging tail posture in order to protect the tail [12,13,14,15].

The pigs in this study were either provided with straw through a rack or directly on the floor, as part of another experiment. It is well known that straw provision has an impact on the development of tail biting (e.g., Schroder-Petersen et al. [2], Wallgren et al. [22]). It is therefore likely that the straw provision affects the occurrence of tail biting, however the association between tail lesions and tail posture is likely not affected. The aim of this study was mainly to investigate the association between tail position and tail damage, which we do not consider to be substantially affected by straw provision. Larsen et al. [13] found a higher probability of having a lowered tail in pigs that were not provided with straw compared to pigs provided with straw; however, the underlying cause for this is not fully understood, and could perhaps be an indicator of the impact of the environment on the emotional state of the pig. As previously discussed, more research is needed to further investigate tail posture in relation to the emotional state of animals [2,12]. Tail biting is multifactorial and has been known also to be affected by, e.g., feeding regime [2]. During this trial, the feeding was altered from four to three meals per day due to the farm normal feeding regimes. This may have affected the occurrence of tail biting. However, all pens experienced the same changes in feeding at the same time, and therefore all pens are likely affected in the same way, and the association between tail lesions and tail posture are assumed to not likely be affected.

The causal relationship between tail posture and tail biting/tail damage is not fully understood. From our study, where tail posture and tail damage was only scored once per week and there was no significant effect of time, it is not possible to discriminate which occurred first, the tail damage or the hanging tail. The causality is therefore unknown. However, Zonderland et al. [11] and Lahrmann et al. [15] found that in weaned piglets hanging tails occurred days before tail damage could be detected. This implies that hanging tails could be an indicator that pigs are trying to protect the tail, although the protection often seems to fail since damage occurs anyway. However, we cannot rule out the possibility that hanging tails are simply more prone to being bitten. According to Kleinbeck and McGlone [12], the tails of pigs that that were being bitten were in the hanging posture. However, as previously mentioned, as observed in both this and other studies, hanging tails are not always damaged [11].

## 5. Conclusions

A hanging tail posture at feeding was found to be significantly correlated to wounds and inflamed wounds on pig tails. Pigs with tail wounds were four times more likely to have hanging tails compared to pigs with undamaged tails. Pigs with inflamed tail wounds were 14 times more likely to have hanging tails compared to pigs with undamaged tails. Only by considering tail position at feeding, 78% of the pigs were able to be correctly classified as having tail wounds or inflamed tail wounds or not. Even though the tail position at feeding is not fully accurate in identifying animals with bitten tails, it is considered feasible in commercial circumstances.

## Figures and Tables

**Table 1:** Descriptive data of tail damage score in relation to tail posture, presented over the production weeks. Tail posture: C = curled tail and H = hanging tail. Pigs for which no observation was made for tail posture or tail damage were omitted from further observations; consequently, ‘Number of obs.’ and ‘No of pigs’ may differ. Pigs were sent to slaughter in weeks 10, 12 and 14.

Week	Tail Posture	Damage					Total	Number of Obs.	Number of Pigs
		Nondamaged	Swollen	Bite Mark	Wound	Inflamed Wound			
1	C	315	41	32	40	1	429	457	459
	H	14	2	2	8	2	28		
2	C	315	41	38	52	3	449	457	459
	H	2			5	1	8		
3	C	281	41	42	50	1	415	451	457
	H	17	2	1	14	2	36		
4	C	252	33	66	58	0	409	449	453
	H	22	4		12	2	40		
5	C	250	46	51	85	3	435	447	452
	H	3	2	1	5	1	12		
6	C	214	47	83	77	4	425	449	452
	H	7	1	4	10	2	24		
7	C	202	42	81	95	3	423	446	450
	H	9	1	2	11		23		
8	C	199	54	64	112	3	432	444	449
	H		2	1	7	2	12		
9	C	238	27	80	79	5	429	445	448
	H	4	1		9	2	16		
10	C	206	66	44	84	3	403	445	447
	H	8	6	5	20	3	42		
11	C	179	47	60	120	5	411	430	435
	H	3		2	12	2	19		
12	C	186	49	46	96	3	380	428	433
	H	7	4	6	28	3	48		
13	C	18	35	40	79	5	337	370	378
	H	5	2	3	20	3	33		
14	C	148	40	49	90	9	336	374	378
	H	6	5	2	17	8	38		

**Table 2:** Tail lesion scoring of tail length, tail damage and wound freshness, adapted from Zonderland et al. [16].

Tail Lesion	Score 0	Score 1	Score 2	Score 3	Score 4
Length	Not shortened	Shortened	Short		
	No shortening	A part of the tail tissue has been bitten off and the tail has been shortened to a length > 2 cm.	A part of the tail tissue has been bitten off and the tail has been shortened to a length < 2 cm.		
Damage	Nondamaged	Swollen	Bite marks	Wound	Inflamed wound
	No visible damage.	The tail is red and/or swollen. The tail has no bite marks and the skin is not broken.	The tail has bite marks that are seen as small red or black dots on the tail. These can either be seen as bruising without broken skin or as small holes in the skin, but no tissue is missing.	The tail has one or more open wounds. These can vary from scratches (without blood) to a shortened tail with a deep wound (with blood). The wound can have a crust that is intact or has partly fallen off.	The tail is swollen and has one or more open wounds. Can vary from scratches (without blood) to a shortened tail with a deep wound (with blood). The wound can have a crust that is intact or has partly fallen off.
Freshness	No blood	Crust	Red crust	Dark blood	Fresh blood
	No blood on the tail. Can be observed together with an open wound (tail damage score greater than “bite mark”) in the case of, for example, small scratches on the tail.	The wound has a crust which is intact or has partly fallen off but where the skin below the crust does not look red or produce any exudate. Can only be observed with an open wound (tail damage score greater than “bite mark”).	The wound has a crust which is intact or has partly fallen off and where the skin below the crust looks red but does not produce any exudate. Can only be observed with an open wound (tail damage score greater than “bite mark”).	There is dark red/brown blood on the tail which feels wet/sticky (i.e., the wound produces exudate), however touching the wound does not lead to a drop of blood on the finger. Alternatively, when the wound has a partly fallen-off crust with the skin below the crust as described above. Can only be observed with an open wound (tail damage score greater than “bite mark”).	Red fresh blood on the tail which feels wet/sticky (i.e., the wound produces exudate), and where touching the wound leads to a drop of blood on the finger. Alternatively, when the wound has a partly fallen-off crust with the skin below the crust as described above. Can only be observed with an open wound (tail damage score greater than “bite mark”).

**Table 3:** Binomial multivariate regression model of hanging tails for 459 pigs in 42 pens over 14 weeks (one observation per week) (n = 6096).

Variables		Odds Ratio (OR)	SE	*p*-Value
**Fixed variables**				
sTime		1.12	1.3	n.s.
sTime^2^		2.09	1.54	n.s.
Damage *(baseline: nondamaged)*	Swollen	1.46	1.28	n.s.
	Bite marks	1.21	1.28	n.s.
	Wound	4.15	1.19	<0.0001
	Inflamed wound	14.24	1.48	<0.0001
Sex *(baseline gilt)*	Barrow	1.58	1.26	0.046
Constant		0.0059	1.36	
**Random variables**				
Pen level	Var (sTime)	1.90	1.34	n.s.
	Var (constant)	1.51	1.26	n.s.
	Cov (sTime, constant)	1.12	1.21	n.s.
Pig level	Var (sTime)	1.40	1.36	n.s.
	Var (sTime^2^)	3.30	2.10	n.s.
	Var (constant)	27.41	2.10	n.s.
	Cov (sTime, sTime^2^)	1.12	1.39	n.s.
	Cov (sTime, constant)	0.99	1.37	n.s.
	Cov (sTime^2^, constant)	0.32	2.03	n.s.

**Table 4:** Tail damage in gilts and barrows (n = 6125).

Sex	Damage				
	0	1	2	3	4
Gilt	1681	317	391	667	36
Barrow	1604	327	419	634	49

**Table 5:** Receiver operating characteristic (ROC) analysis for quantifying the accuracy of tail posture in identifying tail lesions.

Cut-Off Point	Sensitivity	Specificity	Correctly Classified	Positive Likelihood Ratio	Negative Likelihood Ratio
Nondamaged	100	0	6.28	1	
Swollen	70.57	55.38	56.33	1.5816	0.5314
Bite marks	62.76	66.05	65.84	1.8486	0.5638
Wound	55.21	79.62	78.09	2.7089	0.5626
Inflamed wound	8.59	99.16	93.47	10.2516	0.9218
All	0	100	93.72		1

## Supplementary Materials

The following are available online at http://www.mdpi.com/2076-2615/9/1/18/s1: Table S1: Data—kopia.
